# Protozoan Parasites and Type I IFNs

**DOI:** 10.3389/fimmu.2017.00014

**Published:** 2017-01-19

**Authors:** Sasha Silva-Barrios, Simona Stäger

**Affiliations:** ^1^INRS-Institut Armand Frappier, Center for Host-Parasite Interactions, Laval, QC, Canada

**Keywords:** protozoan infections, IFN-I, *Leishmania*, *Toxoplasma*, *Plasmodium*, *Trypanosoma*

## Abstract

For many years, the role of interferon (IFN)-I has been characterized primarily in the context of viral infections. However, regulatory functions mediated by IFN-I have also been described against bacterial infections and in tumor immunology. Only recently, the interest in understanding the immune functions mediated by IFN-I has dramatically increased in the field of protozoan infections. In this review, we discuss the discrete role of IFN-I in the immune response against major protozoan infections: *Plasmodium, Leishmania, Trypanosoma*, and *Toxoplasma*.

## Introduction

Innate and adaptive immune responses are key factors in the control of infectious and chronic diseases; the balance between these two systems is mainly orchestrated by cytokines. Interferons (IFNs) are a large family of cytokines that were first discovered in 1957 in the context of viral infections. The name IFN is due to the capacity of these antiviral factors to interfere with viral replication in mammalian cells (1). Numerous studies have been carried out since their discovery, which allowed the identification of several related molecules. Based on their structural characteristics and the restricted affinity by the receptor molecule with which they directly interact, IFNs are classified into three main groups: type I (IFN-I), type II (IFN-II), and the recently identified type III (IFN-III) (2).

The IFN-I family includes two main classes of related cytokines: IFN-α, which comprises 13 different subtypes encoded by 13/14 different genes; and IFN-β, a product encoded by a single gene and a group of other less studied IFNs (IFN-ϵ, IFNδ, IFNκ, IFNτ, IFNω) (2). The ability to produce and respond to IFN-I is distributed in a wide variety of cells. This confers several autocrine and paracrine effects that have been extensively characterized mainly in viral infections. IFN-I signaling is mediated through a common cell surface receptor, the IFN-I receptor (IFNAR) (3, 4).

The IFN-II family is represented by a single gene product, IFN-γ, and is mainly produced by T lymphocytes and natural killer (NK) cells. IFN-II responses are mediated by the binding of IFN-γ to a heterodimeric molecule, the IFN-γ receptor (IFNGR), ubiquitously expressed in a wide range of cells. IFNGR is involved in the modulation of different cell functions and is a key factor for host defence to intracellular pathogens in various infection models (5).

Finally, the IFN-III family, also known as IFN-λ, comprises four different subtypes: IFN-λ1, IFN-λ2, IFN-λ3, and IFN-λ4. The members of this novel IFN family interact through a unique receptor, the IFN-λ receptor (IFN-λR). In contrast to IFNAR and IFNGR, the expression of IFN-λR is mainly restricted to cells of epithelial origins. The role of IFN-III has yet to be better characterized; however, they appear to induce similar responses to IFN-I (6).

The crosstalk between IFNs and their specific receptors elicits an intracellular signaling cascade that mainly enhances inflammatory responses. The well-characterized signaling cascades of IFN-I and IFN-II are fairly similar. In both cases, Janus kinase 1 (JAK1) and tyrosine kinase 2, associated with IFNAR and IFNGR, are activated. This results in activation and following formation of a heterodimer complex comprised by the cytoplasmic transcription factor signal transducer and activator of transcription 1 and 2 (STAT1/STAT2). STAT1/STAT2 dimers can be translocated to the nucleus and interact with the IFN regulatory factor 9 to form the IFN-stimulated gene factor 3 complex, leading to the transcription of IFN-stimulated genes (ISGs). By contrast, IFN-II signaling through IFNGR activates the JAK/STAT pathway leading to the transcription of pro-inflammatory targets downstream of γ-activated sequences (2, 7).

Interferon (IFN)-I production is mainly induced in response to the activation of receptors on the membrane and/or cytosol, such as pattern recognition receptors (PRRs). PRRs can be activated by conserved pathogens component and endogenous molecules. In most of the cases, the production of IFN-I is related to the activation of PRRs that recognize xenogeneic or autologous nucleic acid, such as toll-like receptors (TLRs) (8).

Interferon (IFN)-I is historically best known for their capacity to elicit antiviral responses; however, they also play a role in bacterial infections and autoimmune diseases (4). The role of IFN-I in regulating the immune response against pathogens is fairly complicated. IFN-I can have enhancing or suppressive effects depending on the disease, the stage of infection, and the amount produced. For instance, IFN-I enhances the antigen-presenting capacity of DCs (9–11), favors the development of T cell responses (12–14), and promotes antibody responses (15, 16) during acute viral infections. By contrast, type I IFNs play an immunosuppressive role during chronic viral infections (17–19), reduce IFN-γ responsiveness in macrophages (20, 21), block B cell functions at high concentrations (22, 23), and can promote the expression of immunosuppressive factors such as IL-10 and PDL-1 (24–27). This duality is also observed in the context of autoimmune diseases, where IFN-I plays a pathogenic role in systemic lupus erythematosus and Sjogren’s syndrome (28, 29), whereas it has therapeutic effects in multiple sclerosis (30).

While IFN-γ has been widely characterized in the modulation of the immune response against protozoan infections, the contribution of IFN-I to host defence against parasites is less clear. In the past few years, a growing body of literature suggests an important role for IFN-I during protozoan infections, particularly in the innate immune response.

In this review, we provide a brief overview of IFN-I mediated effects on the host response in various protozoan infection models and the possible mechanisms involved.

## Protozoan Parasites and IFN-I

Interferon (IFN)-I is involved in the modulation of innate immune responses promoting antigen presentation and NK cell functions. They are also known to play a role in the regulation of the adaptive immune system, promoting the development of antigen-specific T and B lymphocytes against numerous pathogens and inducing immunological memory (7). In most of the cases, these key features are important factors that limit pathogen proliferation; however, IFN-I may also lead to disease exacerbation. Protozoan parasites such as *Plasmodium, Leishmania, Trypanosoma*, and *Toxoplasma* are causing diseases that are among the most lethal and widespread around the world, primarily affecting populations of developing countries. The contribution of IFN-I in the host immune response to these pathogens will be discussed below.

### *Plasmodium* 

*Plasmodium* parasites are the causative agents of malaria, one of the most widespread diseases in the world. The infection presents itself in a wide range of pathologies that can degenerate into severe anemia and the high-risk cerebral malaria (CM). Members of the *Plasmodium* genus have a complex life cycle between an invertebrate (female mosquitoes of the *Anopheles* genus), in which the sexual cycle occurs, and a mammalian host. During the mosquito blood meal, sporozoites are inoculated into the dermis of the mammalian host. In the initial phase of infection, circulating sporozoites can reach lymph nodes, where the priming of B and T cells occurs, or migrate to the liver (31, 32). Within the liver, sporozoites transform first into schizonts within hepatocytes and then into merozoites. This phase is asymptomatic and is known as the pre-erytocytic stage (33). Merozoites are then released into the blood stream. Once they reach the blood, merozoites invade red blood cells, where they undergo cyclic asexual replication initiating the typical symptomatic manifestations of blood-stage malaria, which are caused by the exponential growth of the parasite and massive destruction of erythroid cells (34).

Most of the current knowledge about the immune response to *Plasmodium* parasites has been derived from a combination of *in vitro* and *in vivo* observations in human patients (e.g., *P. falciparum, P. vivax, P. malariae, P. knowlesi*, and *P. ovale*) and murine models of infections (e.g., *P. berghei, P. yoelii, P. chabaudi*, and *P. vinckei*) (34).

During the pre-erythrocytic stage, sporozoite invasion of hepatocytes and subsequent development into merozoites can be blocked by sporozoite-specific antibodies generated by previous exposure to malaria or by immunization; however, this stage is not completely efficient because sporozoites remain in circulation for a short period of time. When T cell priming takes place, infected hepatocytes can be eliminated by cytotoxic CD8 T cells. CD8 T cells, IFNγ, and TNF are critical components required for elimination of infected hepatocytes in humans and the mouse model (35). However, the immune response at this stage is insufficient and released merozoites can reach erythrocytes giving rise to blood-stage malaria (35).

In the erythrocytic stage, early interaction between merozoites and innate immune cells such as dendritic cells, monocytes, macrophages, NK cells, NKT cells, and γδT cells is important for the control of parasite replication and the resolution of infection (33). This phase is characterized by a strong pro-inflammatory response, mediated by the activation of NK, NKT, CD8, and CD4 T cells that produce large amounts of IFNγ and other pro-inflammatory cytokines. IFNγ activates phagocytic cells, such as macrophages, enhancing the secretion of pro-inflammatory cytokines and promoting phagocytosis of circulating parasites and infected red blood cells, which results in the control of parasitemia (36). Polyreactive and specific antibodies against blood-stage malaria can limit parasite propagation between erythrocytes by opsonization and agglutination of parasites and infected erythrocytes; however, humoral responses during the infection are dependent on the presence of circulating merozoites (37). Infected erythrocytes on the surface express parasitic protein which allows them to bind to vascular endothelial cells and avoid clearance. This event induces obstructions in the blood flow and is associated with a strong inflammatory response and the development of CM (33).

Although IFN-γ is the most extensively studied IFN in malaria infection, part of the attention has now been diverted to type I IFNs. IFN-I can have a host-protective or detrimental effect, depending on the stage of the infection or the species of *Plasmodium* involved.

One of the first reports involving type I IFNs demonstrated that administration of mouse serum containing high levels of IFN-I protected mice from *P. berghei* infection by reducing blood parasitemia (38). Similar protective responses were observed after treatment with IFN-β, which prevented death related to CM in *P. berghei*-infected mice (39). By contrast, treatment with recombinant IFN-α during the hepatic cycle in mice infected *P. yoelii* sporozoites did not alter the hepatic parasite burden. However, mice showed reduced parasitemia and decreased signs of immunopathology (40).

*Plasmodium* parasites were reported to induce IFN-I responses. Transcriptomic analysis carried out in mice with blood-stage infection with *P. berghei* revealed that IFN regulatory factors were upregulated during the acute phase (41). Induction of a typical type I IFN signature was also observed in hepatocytes from mice infected with *P. berghei* and *P. chabaudi* sporozoites, where genes such as *Mda, Irf3, Irf7*, and *Stat1* were upregulated (42–44). Similar results were observed in humans. Patients infected with *P. vivax* and *P. falciparum* showed a predominantly IFN-I transcriptional signature during the mild and the severe phase of infection (44, 45).

Recently, Liehl et al. showed that induction of IFN-I during liver stages of the infection is required for host defence against *P. berghei*. Recognition of *P. berghei* nucleic acids by Mda5 induced IFN-I and consequently, the recruitment of leukocytes necessary for parasite elimination in the liver (42). In *P. yoelii*-infected mice, recruitment and expansion of CD49b^+^CD3^+^NKT and CD8^+^T cells to the liver were mediated by IFN-I signaling (43). Migration of neutrophils to the liver is also modulated by IFN-I in mice infected with *P. chabaudi* (44). These studies suggest that functionality of the innate immune response in the liver relies on both IFN-I and IFN-II.

In contrast to the protective effects discussed above, a pathogenic role for IFN-I in *Plasmodium* infections has also been described. For instance, impaired IFN-I signaling has been linked to a protective effect in human patients. Polymorphism in the human gene encoding for IFNAR1 are strongly associated with protection against CM (46). This observation is in agreement with results obtained in a murine model, where the lack of IFN-I signaling led to strong resistance to CM and reduced parasite load during *P. berghei* infection (47, 48). Moreover, in *P. chabaudi*-infected mice, IFN-I appear to suppress Th1 responses that are crucial in the control of hyperparasitemia, by modulating dendritic cell functions (49). In addition, IFN-I and Myd88 signaling are responsible for a decreased recruitment of conventional DCs to the spleen during experimental *P. berghei* or *P. yoelii* infection (50).

Perhaps a better approach for truly understanding the role and function of IFN-I during malaria consists in the identification of modulator molecules that could act in the IFN-I signaling cascade. Recently, regulators of IFN-I response have been identified through genome-wide analysis (Trans-species expression quantitative trait locus, ts-eQLT) during *P. yoelii* infection. Eight genes (*Ak3, Fc*γ*r1, Fosl1, Havcr2, Sipr5, Parp14, Selenbp2*, and *Helb*) had an effect on IFN-I activation. For example, *Fc*γ*r1*^−/−^ mice infected with *P. yoelii* showed significantly higher mRNA and protein levels of IFN-β than wild-type mice, suggesting a negative regulation in the IFN-β response (51).

Future experiments are granted to clarify the spatio-temporal role of IFN-I during malaria.

The role of IFN-I during *Plasmodium* infections is summarized in Table 1.

**Table 1:** **Role of interferon (IFN)-I in *Plasmodium* infection**.

*Plasmodium berghei*	Mouse serum containing high levels of IFN-I	Protection, ↓ blood parasitemia (38)
	Treatment with rIFN-β	Prevents death to cerebral malaria (CM) (39)
	Induction of IFN-1	Required to control hepatic infection (42)
	Lack of IFN-I signaling	↑ Resistance to CM and ↓ parasite load (47, 48)
	IFN-I	↓ Recruitment of conventional DCs to the spleen

*Plasmodium yoelii*	Treatment with recombinant IFN-α	No changes in hepatic burden; ↓ parasitemia and immunopathology (40)
	IFN-I signaling	↑ Recruitment of NKT and CD8 T cells to the liver (43)
	IFN-I	↓ Recruitment of conventional DCs to the spleen

*Plasmodium chabaudi*	IFN-I	↑ Recruitment of neutrophils to the liver (44)
	IFN-I	↓ Protective Th1 responses

### *Toxoplasma* 

*Toxoplasma gondii* is an obligate intracellular protozoan parasite that can infect a wide range of vertebrates and cause a zoonotic disease called toxoplasmosis. *T. gondii* could be considered one of the most successful parasites worldwide; at least 50% of the human population is infected with *Toxoplasma*. The parasite success is mainly due to its ability to invade any nucleated cell and to survive outside the mammalian host (52). *T. gondii* strains are classified in three main lineages, based on the virulence of the strain in the mouse model. This virulence profile does not necessarily correlate to the degree of human infection. Type I strains of *T. gondii* are the most virulent: less than 10 parasites are able to kill a mouse at the onset of infection. By contrast, type II and III strains are less virulent and lead to the establishment of chronic infection (53). *T. gondii* can undergo both asexual (schizogony) and sexual (gametogony) replication. Gametogony and oocyst formation is restricted to feline species that act as a definitive hosts; sexual reproduction of sporozoites occurs within intestinal epithelial cells. Asexual stages of *T. gondii* are not host-specific. Many mammals and birds can act as intermediate hosts. After ingestion of *T. gondii* oocysts by an intermediate host, the parasite transforms into tachyzoites that rapidly undergo multiplication within the parasitophorous vacuole inside various cell types. If the infection is controlled, parasites are retained in tissue cysts; if not, they can cause a systemic lethal disease (54, 55).

Humans are considered as an accidental intermediate host for *Toxoplasma*. In immune-competent individuals, the infection with *T. gondii* is mostly clinically silent, but cause severe diseases in immune-suppressed patients in particular with an impaired T cell and IFNγ response (55). Protective immunity is typically achieved by inducing an IL-12-driven Th1 immune response (56, 57).

In the mouse model, IFN-I can already be detected in the serum of *T. gondii*-infected animals during the acute phase (58–60); IFN-I levels gradually increase with the progression of the infection (60). IFN-I was also detected in the brain and spleen of infected mice (61). These results demonstrate that *T. gondii* not only induces IFN-γ, but also IFN-I.

Recently, inflammatory monocytes were identified as the major source of IFN-β in mesenteric lymph nodes. IFN-β production by inflammatory monocytes required three fundamental events: parasite internalization, TLR activation (mainly TLR4 and 2), and efficient MyD88 signaling. Interestingly, heat killed parasites induced higher levels of IFN-β in inflammatory monocytes (62), suggesting that *Toxoplasma* might limit IFN-I responses (62), possibly by blocking STAT1 (63).

As for many other infection models, the first studies carried out during the 1960s on the role of IFN-I in toxoplasmosis evaluated the impact of a treatment with IFN-I on infected cells *in vitro*. Pre-treatment of mouse fibroblast with IFNs conferred protection to *T. gondii* infection (64). In agreement with this observation, human neonatal and adult macrophages treated with IFN-I were able to control parasite multiplication, even if less effectively than IFN-γ treated cells (65). Moreover, human monocyte-derived macrophages treated with human IFN-β in combination with *Escherichia coli* lipopolysaccharides (LPS), but not with murine IFN-β (MuIFN-β) or rHuIFN-β alone (66), were more resistant to *T. gondii* infection (67).

In the mouse model of toxoplasmosis, treatment with HuIFN-β showed a protective effect, which was enhanced by the combination of rHuIFN-β and LPS and was IFN-γ dependent (66). In agreement with these results, it was shown that *Ifnar*^−/−^ mice orally infected with *T. gondii* have an increased parasite load compared to wild-type mice; higher parasite burdens correlated with a decrease in survival (68).

These results suggest that IFN-β may be produced at the onset of infection to enhance the IFN-γ responses.

A study using human fibroblasts as host cells revealed that treatment of *T. gondii*-infected cells with IFN-I had no effect on parasite replication (69), suggesting that the protective effect of IFN-I depends on cell type and/or timing of exposure to the cytokine (prior to or after infection).

During *T. gondii* infection regulation of tryptophan metabolism is a key component for parasite survival. Indeed, tryptophan degradation inhibits parasite replication. In *T. gondii*-infected mice, indoleamine 2,3-dioxygenase (IDO), a tryptophan catalyzer (70, 71), is enhanced by IFN-II (72). However, it has also been reported that IFN-I can regulate IDO in human retinal pigment epithelial cells, inhibiting therefore *T. gondii* replication (73). Together, these results demonstrate that IFN-I also contribute to the regulation of protective immunity against *T. gondii* (Table 2).

**Table 2:** **Role of interferon (IFN)-I in *Toxoplasma* infection**.

*Toxoplasma gondii*	IFN-I treatment (*in vitro* infection; mouse fibroblasts; and human macrophages)	↑ Resistance to infection (64–67)
	HuIFN-β treatment (*in vitro*)	↑ Resistance to infection (66)
	*Ifnar*^−/−^ mice	↑ Parasite load, ↓ survival (68)
	IFN-I treatment, human fibroblasts	No effects on parasite replication (69)

### *Leishmania* 

*Leishmania* is a complex genus of obligate intracellular protozoan parasites that cause a widespread disease collectively known as Leishmaniasis. The life cycle of these parasites takes place between a mammalian host and a sandfly vector (genus *Lutzomyia* and *Phlebotomus*). Once in the hosts, the promastigote form of the parasite preferentially infects macrophages, but can also be found in other cell types, such as dendritic cells, neutrophils, and fibroblasts. Promastigotes then transform into the non-flagellated form called amastigotes within the host’s cell. The *Leishmania* spp. involved and the mammalian host immune status determine the clinical manifestation of the disease. Parasites can either reside in the skin and/or mucosal surfaces, which results in cutaneous (i.e., *Leishmania major*) or mucocutaneous (i.e., *Leishmania braziliensis*) Leishmaniasis; or disseminate to internal organs such as liver, spleen, and bone marrow, causing visceral Leishmaniasis (VL), the most severe form of the disease (i.e., *Leishmania donovani*) (74).

*Leishmania* immunity is mostly mediated by T lymphocytes. In experimental models, control of infection is mediated by a polarized Th1 response, induced by an initial production of IL-12 by DCs (75). IFN-γ secreting CD4 and CD8 T cells contribute to parasite control by enhancing the ability of phagocytic cells to kill intracellular *Leishmania* (74, 76).

As for many other protozoan models, IFN-II is the main mediators of the cellular immune response. However, IFN-I and IFN-I inducible genes are gradually gaining importance in the *Leishmania* field. One of the pioneer work on the role of IFN-I in Leishmaniasis described the prophylactic treatment with synthetic double-stranded RNA (Poly I:C) prior to *L. donovani* infection. Injection of Poly I:C triggered a burst of IFN-I and led to the control of the hepatic parasite burden (77). The role of endogenous IFN-I was studied for the first time using strains causing cutaneous Leishmaniasis. The induction of IFN-I was observed in macrophages infected *in vitro* with *L. major* promastigotes (78, 79) and in skin macrophages from infected animals (79), showing that promastigotes could enhance IFN-I expression in the host cell. The combination of exogenous IFN-I with *L. major* promastigotes was shown to activate macrophages, inducing type 2 nitric oxide synthase (NOS2). NOS2 is required for parasite elimination; mice deficient in this enzyme are more susceptible to *L. major* infection (80). As for *T. gondii*, the timing of the host cell’s exposure to IFN-I determines the effect on parasite control. Indeed, pre-treatment of macrophages with exogenous IFN-I failed to induce NOS2. Similar results were obtained with high doses of exogenous IFN-I, while a low IFN-I dose in combination with *L. major* enhanced leishmanicidal activity (78, 81). These results suggest that the design of *in vitro* experiments greatly influences the outcome of IFN-I treatment in infected macrophages and that the role of IFN-I should be better studied in *in vivo* models.

The protective role of endogenous IFN-I during infection was confirmed by neutralizing IFN-I in mice experimentally infected with *L. major*. In fact, IFN-I neutralization rendered *L. major*-infected mice more susceptible to infection and enhanced parasite multiplication. IFN-I blockade led to abolishment of NOS2 function and reduced cytotoxic activity and IFNγ production by NK cells at early stages of infection (79).

Opposite results were obtained in human macrophages infected *in vitro* with New World *Leishmania* spp. IFN-β treatment of *L. braziliensis* and *Leishmania amazonensis*-infected macrophages enhanced the parasite burden through a superoxide-dependent, NO-independent mechanism (82). In this model, it was shown that IFN-β can regulate the superoxide dismutase SOD1 activity. SOD1 is responsible for catalyzing the disproportionation of superoxide to hydrogen peroxide and dioxygen and is an important constituent in apoptotic signaling and oxidative stress. It has been observed that biopsies from cutaneous Leishmaniasis patients express high levels of SOD1 (82).

The importance of endogenous IFN-I during chronic infection has been investigated using IFNAR-deficient mice in the context of *L. amazonensis* infection. *L. amazonensis* infected *Ifnar*^−/−^ mice developed attenuated cutaneous lesions and displayed a decreased parasite load. This effect appeared to be STAT1 independent, a key protein in the IFN signaling (83). Furthermore, *L. amazonensis*-infected *Ifnar*^−/−^ mice exhibited high levels of neutrophils and lower inflammatory monocytes recruitment at early times post infection. This unique profile was also observed in *L. major* and *L. braziliensis* infections (83). *In vitro* coculture of infected WT macrophages with *Ifnar*^−/−^ neutrophils revealed that IFNAR-deficient neutrophils promote parasite killing (83). This evidence supports the pathogenic role of IFN-I signaling in cutaneous Leishmaniasis caused by New World *Leishmania* species.

We also observed a negative role for IFN-I in an experimental model of VL. *L. donovani* amastigotes were shown to induce IFN-I expression in B cells in an endosomal TLR-dependent manner. This cytokine was involved in a positive regulatory loop that led to the upregulation of endosomal TLRs and to IL-10 production in B cells (84). B cell-derived IL-10 was shown to suppress protective T cells responses and increase disease susceptibility (85). B cells are known to play a detrimental role during VL (86), not only by secreting IL-10 but also for their excessive antibody production (87). Indeed, hypergammaglobulinemia is a hallmark of VL. Interestingly, IFN-I seems to be regulating antibody production during VL. Specific ablation of endosomal TLRs or IFN-I signaling in B cells was shown to severely reduce the Ig titer in the serum of *L. donovani*-infected mice, suggesting that parasite activation of B cells *via* endosomal TLRs and IFN-I are involved in the induction if hypergammaglobulinemia (84). Furthermore, mice with a B cell-specific deficiency in endosomal TLR or IFNAR were more resistant to *L. donovani* infection than their wild-type counterpart.

Very little is known about the function of IFN-I in VL patients. It was reported that human mononuclear phagocytes can be activated by IFN-β, but less efficiently than IFN-γ (88). Exogenous treatments with IFN-I and IFN-II but not IL-2, failed to restore the cytotoxic activity of NK isolated from VL patients (89). Also, treatment of the cutaneous lesion in patients with IFN-I did not improve healing, compared with IFN-γ treatment (72, 90).

Because dendritic cells can also be infected by *Leishmania*, it is important to consider the induction of IFN-I by the parasite and its possible effect in these cells as well. Transcriptomic analysis of human DCs infected *in vitro* with *L. major* or *L. donovani* showed a differential expression pattern for IL-12 associated genes, the NF-KB pathway, and IFN regulatory factors (91). IFN-β produced by *L. major*-infected DCs seems to be required for IL-12 secretion by the infected DC, suggesting that protective Th1 responses, which are IL-12 depended, may also depend on IFN-I (92).

A summary on the role of IFN-I during Leishmaniasis can be found in Table 3.

**Table 3:** **Role of interferon (IFN)-I in *Leishmania* infection**.

*Leishmania donovani*	Treatment with Poly I:C	↓ Hepatic parasite burden (77)
	B cell-derived IFN-I	↑ IL-10, ↑ hypergammaglobulinemia (84)

*Leishmania major*	IFN-I treatment of macrophages *in vitro* (78–81)	
	(1) At the time of infection (2) Before infection (3) High dose (4) Low dose *In vivo* IFN-I blockade	↑ NOS2 No effect on NOS2 No effect ↑ Leishmanicidal activity ↓ NOS2, ↓ natural killer functions (79)

*Leishmania braziliensis*	IFN-β treatment of macrophages *in vitro*	↑ Parasite burden (82)

*Leishmania amazonensis*	*Ifnar*^−/−^ mice	↓ Lesions, ↓ parasite burden, ↑ neutrophils (83)

### Trypanosomes

Trypanosomes are digenetic protozoan parasites that infect domestic and wild animals, as well humans. Although many species of trypanosomes cause important veterinary disease, mainly two species cause significant human morbidity: *Trypanosoma brucei* and *Trypanosoma cruzi*. These two species are responsible for causing the sleeping sickness (African trypanosomiasis) and the Chagas disease (American trypanosomiasis), respectively.

The life cycle of these parasites takes place between the invertebrate vector and the vertebrate host. *T. brucei* and other African’s trypanosomes are transmitted to the mammalian host by a tsetse fly bite. In the blood stream, metacyclic trypomastigotes differentiate into bloodstream trypomastigotes. In humans, trypanosomes proliferate in the blood and lymphatic system at early stages of the infection. This stage is associated with an anti-inflammatory response. At chronic stages, parasites can pass through the blood–brain barrier and enter the central nervous system. This stage is associated with inflammatory changes in the brain and is characterized by a neurological disturbance (93, 94).

In *T. cruzi* (American trypanosomiasis), metacyclic trypomastigotes are released in the feces/urine of the triatomine vector after a blood meal. Trypomastigotes can successfully infect the mammalian host if they are able to reach the mucosa or injured skin areas. In contrast to African trypanosomes, *T. cruzi* is an intracellular parasite that has the capacity to invade, differentiate into amastigotes, and replicate within a wide range of nucleated cells. This characteristic is one of the most important features of *T. cruzi* within the host. Amastigotes differentiate into infective bloodstream trypomastigotes, before being released upon cell lysis. The released parasites can then infect neighboring cells or enter the bloodstream (95).

During the acute phase, the innate immune response against *T. cruzi* is characterized by the induction of a cell-mediated response that involves the production of IFN-γ and TNF (by NK and T cells), required for enhancing iNOS activity by phagocytic cells and for priming the adaptive immune response. iNOS activation is critical for controlling parasite growth during the infection (95). *T. cruzi* elicits a prominent IFN-I response at early times of infection (96–99). As mentioned before for *Plasmodium*, the role of IFN-I in *T. cruzi* infection is controversial. Some studies ascribe a protective role to IFN-I; others demonstrate that IFN-I induces pathology. The effect of IFN-I mainly depends on the dose, amount of parasites, and the inoculation route used to set up the infection.

The first studies on the role of IFN-I investigated the outcome of exogenous IFN-I treatment in *T. cruzi*-infected mice. The results showed that administration of IFN-I increased resistance to infection by stimulating T and NK cell activities, which are essential for protection (100, 101).

In an intradermal model of infection, transcriptomic analysis of excised skin from the inoculation site revealed that *T. cruzi* upregulated the expression of ISGs as early as 24 h after infection. Induction of ISGs was dependent on IFN-I signaling, suggesting that IFN-I is an important component of the innate immune response to *T. cruzi* (99). In agreement with the above mentioned literature, studies carried out in *Ifnar*^−/−^ mice infected with *T. cruzi* revealed that efficient IFN-I signaling was required for controlling parasites growth during the acute phase of infection (102, 103). IFN-I was necessary for enhancing NO production in phagocytic cells (102). NO is considered the major effector molecule for intracellular amastigotes elimination within infected cells, being important for the control of parasite multiplication (95).

By contrast, another group reported a potential pathogenic role for IFN-I. In this work, a lethal dose of parasites inoculated intradermally was used to set up the infection in WT and *Ifnar*^−/−^ mice. Surprisingly, *T. cruzi*-infected *Ifnar*^−/−^ mice survived the challenge and were able to control parasite replication (104). Besides the fact that splenocytes from *Ifnar*^−/−^ mice produced higher levels of IFN-II, plasma cytokine profile in *T. cruzi*-infected *Ifnar*^−/−^ mice were not different to control mice (104). Additionally, T cells populations were not inherently different compared with control mice (104), and IFN-γ production by CD8^+^T cells was not affected by impaired IFN-I signaling (105), suggesting that, in this model, endogenous IFN-I is not the only relevant signal in host defense against *T. cruzi*.

Taken together, the role of IFN-I in *T. cruzii* infection differs from one experimental model to the other, depending on the dose and the route of infection (106). This could explain the controversy about the observations on the role of IFN-I in the *T. cruzi* model of infection (Table 4).

**Table 4:** **Role of interferon (IFN)-I in *Trypanosoma* infection**.

*Trypanosoma cruzi*	IFN-I treatment *in vivo*	↑ Resistance to infection
		↑ T and natural killer cell activity (100, 101)
	*Ifnar*^−/−^ *mice*	Disease exacerbation (102, 103)
	*Ifnar*^−/−^, lethal dose	↑ Survival (104)

*Trypanosoma brucei rhodesiense*	*Ifnar*^−/−^, acute phase	↑ Control
	*Ifnar*^−/−^, later stages	↓ Resistance, IFN-γ ↓ (108)

*Trypanosoma brucei brucei*	*Ifnar*^−/−^	No effect on parasite control (109)

The immune response to African trypanosomes is quite different than that to *T. cruzi*. First, parasites never enter the host cell at any stage of their development. The success of these parasites is mainly due to their ability to change the composition of the variant surface glycoprotein (VSG) by switching genes. This confers them the capacity to evade B- and T-cell-mediated immune responses and results in fluctuating waves of parasitemia that characterize African trypanosomiasis (94). VSG is a strong antigen that induces Th1 responses and promotes autoantibody and cytokines production, in particular TNF. Other trypanosome proteins and soluble factors, such as a trypanosome-released triggering factor, also trigger IFN-γ production by T and NK cells and are involved in macrophage activation toward an M1 phenotype, which is required for the control of parasite multiplication during the acute phase of infection. However, sustained activation of M1 macrophages is associated with disease exacerbation. The progression of the infection toward the development of an acute fatal disease or a prolonged chronic infection is determined by the balance between a type I and type II immune responses and the switch from the early type I immune response (dominated by M1 macrophage activation) from a type II (M2 macrophages) regulatory response that controls the inflammation (107).

The literature on the role of IFN-I in African trypanosomiasis is scarce. A study involving *Trypanosoma brucei rhodesiense* reported a beneficial effect of IFN-I during the acute phase of infection. Indeed, *Ifnar*^−/−^ mice displayed delayed control of parasite burden during the first week of infection and died earlier than wild-type controls. Moreover, mice hyperresponsive for IFN-I (*Ubp43*^−/−^) exhibited a significant defect in Th1 responses and IFN-γ production, suggesting that IFN-I plays a role in the early stages of disease. Nevertheless, IFN-I contributes to the downregulation of IFN-γ production and loss of host resistance during chronic infection (108).

No effects of IFN-I signaling were observed in *Trypanosoma brucei brucei*-infected *Ifnar*^−/−^ mice, which showed similar levels of parasitemia to wild-type mice, suggesting that in this model parasite control is independent of IFN-I (109). However, IFN-I regulates T cell infiltration to the brain parenchyma at chronic stages of the infection (109).

In conclusion, the contribution of IFN-I to protective immunity against several protozoan parasites is still unclear. Variations in parasite numbers used for infections, the site of inoculation, and the dose of IFN-I all seem to influence the outcome and the interpretation of the results. A spatio-temporal analysis of the role of IFN-I integrated with a more detailed investigation of cell-specific signaling pathways elicited by the cytokine could help to better dissect the involvement of IFN-I in the immune response.

## Author Contributions

All authors listed have made substantial, direct, and intellectual contribution to the work and approved it for publication.

## Conflict of Interest Statement

The authors declare that the research was conducted in the absence of any commercial or financial relationships that could be construed as a potential conflict of interest.
